# State-of-the-Art Sensors Research in Ireland

**DOI:** 10.3390/s22020629

**Published:** 2022-01-14

**Authors:** John Barton, Mark Ferguson, Cian Ó Mathúna, Elfed Lewis

**Affiliations:** 1 Micro/Nano Systems Centre, Tyndall National Institute, University College Cork, Dyke Parade, T12 R5CP Cork, Ireland; cian.omathuna@tyndall.ie; 2 Science Foundation Ireland, Three Park Place, Hatch Street Upper, D02 FX65 Dublin, Ireland; markwjferguson@btinternet.com; 3 School of Engineering, University College Cork, Lee Maltings, Dyke Parade, T12 R5CP Cork, Ireland; 4 Optical Fibre Sensors Research Centre (OFSRC), University of Limerick, V94 T9PX Limerick, Ireland; Elfed.Lewis@ul.ie

## 1. Introduction

This Special Issue captures a significant portion of the current sensors research excellence in Ireland. Through the normal MDPI journal review process, the editors have selected 28 publications from 164 authors with a 1:2.5 female to male ratio from 11 Higher Education Institutions and 9 companies across the entire island of Ireland.

In line with the common prediction “Everything that can be measured will be sensored, everything that is sensored will be connected and everything that is connected will be automated”, this review provides a timely snapshot of the incredible breadth of applications and potential impact of sensors on health, wellbeing, security, energy, the environment, food production, supply chain, transport and manufacturing.

From my perspective, an interesting aspect of this Special Issue is the level of input from SFI (Science Foundation Ireland)-funded research, which illustrates the strategic impact that SFI [1] has had on the development and growth of Ireland’s national research priorities, and its international profile in sensor research.

There are 12 publications from the five most relevant SFI Research Centres: INSIGHT [2], LERO [3], CONNECT [4], CONFIRM [5] and VISTAMILK [6]. A further four publications are affiliated with an SFI Career Development Award, SFI TIDA (Technology Innovation Development Award) and a Royal Society/SFI University Research Fellowship. It is also impressive to see industry co-authors from multinational companies such as Alcon Laboratories, DP Energy Ireland, Johnson & Johnson, P&O Maritime Logistics and ZfB; SMEs (small and medium enterprises) including Aerogen, Green Rebel Marine, and other organisations including Beacon Hospital and No Barriers Foundation.

Research Excellence in Ireland is distributed across all of the Higher Education Institutions and companies in Ireland, and many SFI programmes such as Research Centres, Spokes, and Partnerships, foster such collaboration. These programmes have had a major impact; over the last 10 years the number of industry collaborations has increased from 702 to 1715; the amount of industry co-funding of that research has increased from EUR 8 million to EUR 93 million per annum; and the number of SFI-funded Industry Research and Innovation Fellowships increased from 0 to 200. This enhanced level of industry–academic research collaboration has also contributed to Ireland’s increased research standing, with the number of highly globally cited researchers increasing in the same time period from 5 to 26, and Ireland’s performance in the European R&I programme, Horizon 2020, doubling from c. EUR 600 million to EUR 1.25 billion.

I am delighted to see the outputs of this research funding and collaboration in the important field of sensor research reflected in the papers in this Special Issue, and congratulate the editors and authors for putting this together.

***Professor Mark WJ Ferguson***

***Director General, Science Foundation Ireland and Chief Scientific Adviser to the Government of Ireland***

***December 2021***

## 2. Summary of Papers in Special Issue

Following a rigorous peer review process, 28 papers were accepted for this Special Issue, and while representing the diverse nature of sensor research in Ireland, we have broadly grouped them into four categories: (1) Micro and Nano Sensors, (2) Sensor Systems: Signals, Processing, and Interfaces, (3) Sensor networks and Smart/Intelligent sensors, and (4) Applications. Many of the submissions may overlap and exist in more than one of these categories.

### 2.1. Micro and Nano Sensors

In [7], Cavaliere et al. propose a planar inductive sensor design, printed on PCB and embedded into medical patches, for electromagnetic tracking in image-guided interventions. Electromagnetic tracking is a safe, reliable, and cost-effective method to track medical instruments in image-guided surgical navigation. Large-area planar sensors show high sensitivity, even in the absence of a magnetic core. They can be printed on flexible PCB, and embedded in adhesive patches, with reduced costs and high-precision manufacturing and repeatability. This paper analyses the tracking performances of planar coils on the centimetre scale. An accurate magnetic model is proposed and experimentally validated.

Seymour et al. explore the use of micro- and nano-scale electrode devices in [8] for the development of an electrochemical sensing platform, to digitalize a wide range of applications within the agrifood sector. With the advancement of electrode modification techniques, along with tailored electrochemical analysis, these devices can address many of the sensing requirements associated with the emerging agricultural 4.0 industry. A portable electrochemical reader system is presented, capable of performing the desired analysis on-farm. The significance of this is that the turnaround time between obtaining an analytical result and responding to any issues can be completed on-site in a matter of minutes.

Electrochemical nanosensors are also the focus of [9] by Wang et al., who review the operation of electrochemical cells and propose an equivalent electric circuit model for electrochemical nanosensors. The proposed model extends the operation of the well-established Randles model beyond a two-electrode electrochemical sensor to a three-electrode electrochemical sensor. In doing so, the proposed model provides an equivalent circuit that can, for the first time, be an EIS-based integrated system design. A comparison of the two- and the three-electrode model is presented, and a fitting simulation is conducted to show the feasibility of the application of three-electrode electrochemical sensors in interface circuits design and electrochemical impedance analysis.

McGuinness et al. [10], present a comparison of an extrinsic Fabry–Perot interferometer (EFPI)-based temperature sensor, constructed using a novel diaphragm manufacturing technique, with a reference all-glass EFPI temperature sensor. The novel diaphragm was manufactured using polyvinyl alcohol, and sensor fabrication involved fusing a single-mode fibre to a length of fused quartz capillary, which has an inner diameter of 132 μm and a 220 μm outer diameter. Both temperature sensors were placed in a thermogravimetric analyser and heated from an indicated 30 to 100 °C to qualitatively compare sensitivities. Initial results indicated that the novel manufacturing technique both expedited production and produced a more sensitive sensor when compared to an all-glass construction.

Nagle et al. [11] present the development of a nanoporous gold (NPG) electrochemical sensor, capable of creating a unique fingerprint signal generated by inhalable pharmaceuticals, which provided the impetus for a study on the electrooxidation of salbutamol, the active bronchodilatory ingredient in Ventolin^TM^ formulations. It was demonstrated that, in NPG-modified microdisc electrode arrays, salbutamol is distinguishable from the chloride excipient present at 0.0154 M using linear sweep voltammetry, and can be detected amperometrically. In contrast, bare-gold microdisc electrode arrays cannot afford such discrimination, as the potential for salbutamol oxidation and chloride adsorption reactions overlap.

In [12], Daly et al. report on the fabrication and characterization of platinum-based interdigitated microband electrode arrays and their application for trace detection of copper. Using square-wave voltammetry after acidification with mineral acids, a limit of detection of 0.8 μg/L was achieved. Copper detection was also undertaken on river water samples and compared with standard analytical techniques. The possibility of controlling the pH at the surface of the sensors—thereby avoiding the necessity to add mineral acids—was investigated. Detection of standard copper solutions down to 5 μg/L (ppb) using this technique is reported.

### 2.2. Sensor Systems: Signals, Processing, and Interfaces

Walsh et al. [13] investigated the coating evaluation of solid lubricant coatings on concealed/disjointed surfaces for transparent polymer delivery device applications. An IR transmission microscope set-up, which is automated to measure eight nozzles in ≈3 min, was constructed to measure coating coverage. A test was developed to replace the standard industrial dye-stain technique for identifying deficient coating. Inferior coating processes were used to produce consignments of samples with dye-stain fail rates between 5% and 100%. The two testing techniques had, at times, large discrepancies in their fail rates, with that of the IR test generally being higher.

In [14], Wang et al. described the development of a driving simulator platform that includes a synchronous recording of eye tracking, EEG, and driver motor response. The apparatus was used to record data from participants viewing static and dynamic billboards. The cognitive responses generated by the stimuli were extracted from the data. The analysis shows increased cognitive activity related to dynamic billboards. The level of engagement with the billboard is likely to be a precursor to driver distraction. This paper has shown that it is possible to analyse this step in the distraction process, and demonstrates the functionality of the proposed measurement system as a valid tool in assessing driver cognitive responses to billboards.

Argent et al. [15], aimed to conduct a thorough evaluation of an example wearable exercise biofeedback system from laboratory testing through to clinical validation in the target setting, illustrating the importance of context when validating such systems. The results show a reduction in overall system accuracy between lab-based cross validation (>94%), testing on healthy participants (*n* = 10) in the target setting (>75%), through to test data collected from the clinical cohort (*n* = 11) (>59%). This study illustrates that the reliance on lab-based validation approaches may be misleading key stakeholders in the inertial sensor-based exercise biofeedback sector.

Peres et al. [16] present the development of a novel, miniature, low-power, NFC-enabled data acquisition system to monitor seaweed growth parameters in an aquaculture context. It logs temperature, light intensity, depth, and motion, and these data can be transmitted or downloaded to enable informed decision making for seaweed farmers. The device is fully customisable and designed to be attached to seaweed or associated mooring lines. The developed system was characterised in laboratory settings to validate and calibrate the embedded sensors. It performs comparably to commercial environmental sensors, enabling the use of the device to be deployed in commercial and research settings.

Barton et al. [17], demonstrate how convolutional neural networks may be enhanced with a nonstandard loss function in order to improve the overall signal-to-noise ratio of Raman spectra, while limiting corruption of the spectral peaks. Simulated Raman spectra and experimental data are used to train and evaluate the performance of the algorithm in terms of the signal-to-noise ratio and peak fidelity. The proposed method is demonstrated to effectively smooth noise while preserving spectral features in low-intensity spectra, and was shown to improve the SNR by up to 100% in terms of both local and overall SNR.

In [18], Vijayan et al. give a review of wearable devices and data-collection considerations for connected health. This review examined various wearable devices used for quantified self, clinical assessments and automated monitoring of activity and sleep patterns. Current studies employing deep-learning approaches to automatically detect certain activity or sleeping patterns from data received from wearable technologies are also examined.

### 2.3. Sensor Networks and Smart/Intelligent Sensors

The feasibility of equine, field-based, postural sway analysis using a single inertial sensor was investigated by Egan et al. [19]. This is the first study to investigate how often and for how long equines settle into a state of quiet standing, which would enable the analysis of postural sway captured in the applied setting. The authors submit that conclusions on the health of the sensorimotor system based on postural sway depends both on the context in which it is measured (e.g., motor development, neurological disease, and limb injury), the particular metric of postural sway that is analysed, and whether or not it is in a laboratory- or field-based environment.

A study by Henderson et al. [20] proposed a state-of-the-art measurement system for monitoring data glove technologies. The system was designed to have high precision and compactness, as well as to allow for the total functionality of the moving arm. In addition, the system was designed to mimic finger-joint movement from hyper extension to full flexion. All data gloves/sensors were verified under dynamic conditions because they must be able to record the parameters of a finger’s continuous motion in most applications. To verify the accuracy, reliability, and stability of the system, two verification tests were set and conducted.

Data gloves were also the focus of another paper by Henderson et al. [21], but this time concentrating on a review of wearable, sensor-based, health-monitoring glove devices for Rheumatoid Arthritis (RA). This paper provides an updated review among the sensor and glove types proposed in the literature to assist with the diagnosis and rehabilitation activities of RA. Consequently, the main goal of this paper is to review contact systems and to outline their potentialities and limitations.

Dhirani et al. [22] discuss various cyberthreats and risks which the Industrial IoT (IIoT/I4.0) is exposed to, regardless of implementing cybersecurity standards and security protocols. IIoT/I4.0 is a fully connected autonomous factory which requires interoperable and universal standards, bringing convergence and mitigating the security gaps that exist between the standards and controls. Each smart factory may have different service and security requirements, and requires a customized security strategy solution aligning different cyber standards mitigating the threat landscape. The unified roadmap designed by the authors contributes toward (i) securing the heterogenous production environment, (ii) providing guidelines for identifying, assessing, and mitigating novel cybersecurity-based threats, (iii) implementing different levels of protection, and (iv) providing IT/OT convergence and alignment.

A review by O’Mahony et al. [23] focuses on the state-of-the-art methods, applications, and challenges of representation learning for fine-grained change detection. Fine-grained change detection in sensor data is very challenging for artificial intelligence, though it is critically important in practice. It is the process of identifying differences in the state of an object or phenomenon where the differences are class-specific and are difficult to generalise. As a result, many recent technologies that leverage big data and deep learning struggle with this task. This research focuses on methods of harnessing the latent metric space of representation learning techniques as an interim output for hybrid human–machine intelligence.

The paper by Gawade et al. [24] reports on a smart museum archive box that features a fully integrated, wireless powered temperature and humidity sensor. The smart archive box has been specifically developed for microclimate environmental monitoring of stored museum artifacts in cultural heritage applications. The developed sensor does not require a battery and is wirelessly powered using Near Field Communications (NFC). The proposed solution enables a convenient means for wireless sensing with the operator by simply placing a standard smartphone in close proximity to the cardboard archive box.

### 2.4. Applications

In [25], McDevitt et al. describe a free-living and supervised protocol comparison study of the Verisense inertial measurement unit to assess physical activity and sleep parameters and compares it with the Actiwatch 2 actigraph. It is a pertinent challenge to find a single reliable sensor to assess both physical activity and sleep while adhering to budgets and maximising participant compliance through minimising burden. Overall, the results showed moderate-to-high agreement of Verisense with the Actiwatch 2 for assessing epoch-by-epoch physical activity and sleep, but a lack of agreement for activity classifications. Future validation work of Verisense for activity cut-point potentially holds promise for 24 h continuous remote patient monitoring.

The paper by Madden et al. [26] examines the current state of the art of commercially available outdoor footfall sensor technologies, and defines individually tailored solutions for the walking trails involved in an ongoing research project. Effective implementation of footfall sensors can facilitate quantitative analysis of user patterns, inform maintenance schedules and assist in achieving management objectives, such as identifying future user trends such as cyclotourism. It incorporates the footfall-capture and management experiences of trail management within the EU Atlantic area and desk-based research on current footfall technologies and data-capture strategies.

Yeong et al. [27] present a review of sensor and sensor-fusion technology in autonomous vehicles. Sensors are fundamental to the perception of vehicle surroundings in an automated driving system, and the use and performance of multiple integrated sensors can directly determine the safety and feasibility of automated driving vehicles. This paper evaluates the capabilities and the technical performance of sensors which are commonly employed in autonomous vehicles, primarily focusing on a large selection of vision cameras, LiDAR sensors, radar sensors and the conditions in which such sensors may operate in practice. The paper, therefore, provides an end-to-end review of the hardware and software methods required for sensor-fusion object detection.

Duddy et al. [28] examined the effects of powered exoskeleton gait training on cardiovascular function and gait performance via a systematic review. The included studies indicated that powered exoskeleton-assisted training may increase oxygen consumption to a level similar to non-exoskeleton walking, and elevate HR to a greater level than non-exoskeleton waking. Therefore, powered exoskeleton gait training may be an effective way to improve cardiovascular function and walking ability for spinal cord injury, multiple sclerosis and stroke patients. The powered exoskeleton appears to be a novel method of facilitating paraplegic individuals to achieve physical activity guidelines for health recommended by the World Health Organisation.

The “Little MonSta” benthic lander array described by Wheeler et al. [29] consists of eight ROV-deployable (remotely operated vehicle) instrumented lander platforms for monitoring physical and chemical oceanographic properties and particle sampling, developed as part of the MMMonKey_Pro program (mapping, modelling, and monitoring key processes and controls in cold-water coral habitats in submarine canyons). A proof-of-concept case study was presented from the cold-water coral habitable zone in the upper Porcupine Bank Canyon, where the Little MonStas collected 868.8 h of current speed, direction, temperature, and benthic particulate flux records, as well as 192 particle samples subsequently analysed for particular organic carbon (POC), lithic sediment, live foraminifera, and microplastics.

Khaki et al. [30] examined particulate matter (PM) emissions generated by 3D printers in an indoor domestic setting. Significant impacts on PM emissions were observed for many of the parameters investigated, whereby the filament brand and colour, for example, can dramatically influence the PM emission and hence influence personal exposures and ultimately user health. To give users an awareness of PM emissions during their own printing activities, low-cost indoor air-quality PM sensors have been demonstrated here as a viable way to monitor PM emissions during printing, which can alert the home user when increased ventilation is needed.

The design and construction of an aluminium coil array for electromagnetic tracking applications was presented by O’Donoghue et al. [31]. The accuracy of this new field generator has been shown to be 1.5 mm RMS, which was only marginally worse in comparison to the standard coil arrays used with the system. From a comparison of two different X-ray imaging modalities, the low X-ray absorption of the aluminium coils was clearly demonstrated. The creation of effectively radiolucent electromagnetic tracking systems allows the usage of the technology to be applied to a wide range of different procedures where interference with the imaging modalities used is a major concern.

Kelly et al. [32] discussed the importance of accurate and objective clinical balance-assessment methods. A review was conducted on the state of the art related to technology-enabled balance-assessment methods and their potential to be utilised in remote rehabilitation settings. A set of six key themes were identified and discussed. Results of the literature review led to the creation of an objective assessment rubric based on four scoring components comprising a total of nine assessment criteria. Each criterion was identified based on its importance in real-world deployment of remote rehabilitation technology, and a weighting to each criterion was decided by three independent physiotherapists.

Behera et al. [33] presented a systematic review of state-of-the-art sensors for remote care of people with dementia during a pandemic. This review presented existing assistive technology (AT) for people living with dementia (PLWD), and provided the pros and cons of the existing technical solutions for dementia patients that might be helpful during a pandemic which enforces restrictions on physical contact. The main aspects of clinical trials conducted to study the utility of AT devices for dementia are also presented, along with important requirements for future directions in these areas of research which can improve the effectiveness of devices for PLWD and their carers.

In [34] by Olusoji et al., the effect of low doses of X-ray and proton radiation on phosphorus-doped fibres was investigated for possible use in dosimetry applications. The study indicates that the radiation-induced alteration in glass properties results in RIA for all different particles and energies tested, as expected based on known interactions of particles with matter. The RIA in the fibre for both the proton and X-ray beam is linear within the dose delivered. Overresponse of the fibre in the Bragg peak of the proton beam suggests that the fibre is too sensitive for long irradiations at high LET, especially for wavelengths with higher sensitivity, because of the saturation effect; wavelengths with lower sensitivity are better suited for dosimetry applications.
